# Effects of Alkoxy and Fluorine Atom Substitution of Donor Molecules on the Morphology and Photovoltaic Performance of All Small Molecule Organic Solar Cells

**DOI:** 10.3389/fchem.2018.00413

**Published:** 2018-09-13

**Authors:** Beibei Qiu, Shanshan Chen, Lingwei Xue, Chenkai Sun, Xiaojun Li, Zhi-Guo Zhang, Changduk Yang, Yongfang Li

**Affiliations:** ^1^CAS Key Laboratory of Organic Solids, CAS Research/Education Center for Excellence in Molecular Sciences, Institute of Chemistry, Chinese Academy of Sciences, Beijing, China; ^2^School of Chemical Science, University of Chinese Academy of Sciences, Beijing, China; ^3^Department of Energy Engineering, Low Dimensional Carbon Materials Center, School of Energy and Chemical Engineering, Ulsan National Institute of Science and Technology, Ulsan, South Korea; ^4^Laboratory of Advanced Optoelectronic Materials, College of Chemistry, Chemical Engineering and Materials Science, Soochow University, Suzhou, China

**Keywords:** benzothiadiazole, organic small molecule donors, fluorine substitution, alkoxy side chain, all small molecule organic solar cells

## Abstract

Two benzothiadiazole (BT)-based small-molecule donors, SM-BT-2OR with alkoxy side chain and SM-BT-2F with fluorine atom substitution, were designed and synthesized for investigating the effect of the substituents on the photovoltaic performance of the donor molecules in all small molecule organic solar cells (SM-OSCs). Compared to SM-BT-2OR, the film of SM-BT-2F exhibited red-shifted absorption and deeper HOMO level of −5.36 eV. When blending with *n*-type organic semiconductor (*n*-OS) acceptor IDIC, the as-cast devices displayed similar PCE values of 2.33 and 2.76% for the SM-BT-2OR and SM-BT-2F-based devices, respectively. The SM-BT-2OR-based devices with thermal annealing (TA) at 120°C for 10 min showed optimized PCE of 7.20%, however, the SM-BT-2F-based device displayed lower PCE after the TA treatment, which should be ascribed to the undesirable morphology and molecular orientation. Our results reveal that for the SM-OSCs, the substituent groups of small molecule donors have great impact on the film morphology, as well as the photovoltaic performance.

## Introduction

Organic photovoltaics (OPV), as one of the most promising next generation technologies to utilize solar energy, have been extensively investigated during the past several decades, due to its attractive advantages of light-weight, low-cost and capability to be fabricated into flexible and semitransparent devices (Liang and Yu, 2010; Li, 2012; Li et al., 2012). In recent years, bulk-heterojunction (BHJ) organic solar cells (OSCs) that use small-molecule *n*-type organic semiconductor (*n*-OS) as acceptor have gained significant progress (Lin and Zhan, 2014; Nielsen et al., 2015). In particular, most recently, power conversion efficiencies (PCEs) over 14% for single-layer device and 15% for tandem device have been achieved, demonstrating great potential for the commercialization of OSCs (Che et al., 2018; Li et al., 2018; Zhang et al., 2018).

The rapid development of OSCs is a combination of the innovation of donor and acceptor photovoltaic materials, the methodology of morphology tuning and the optimization of device structures (Lee et al., 2008; Hau et al., 2010; Huang et al., 2014; Wang and Kyaw, 2014; Ye et al., 2014; Gao et al., 2015; Lin and Zhan, 2016; Zhang et al., 2016; Yan et al., 2017). Especially, the progress of photovoltaic materials plays a critical role in promoting the development of OSCs (Nielsen et al., 2015; Zhang and Zhu, 2017; Wadsworth et al., 2018). Especially, great improvement of the OSCs has been achieved by the development of wide bandgap conjugated polymer donors and narrow bandgap *n*-OS small-molecule acceptors (Bin et al., 2016a; Sun et al., 2018). Although small-molecule *p*-type organic semiconductor (*p*-OS) donors, compared with polymer donors, possess the advantages of well-defined chemical structures and easy purification, the photovoltaic performance of the all small molecules OSCs (SM-OSCs) is relatively lag behind, because of its more difficult morphology tuning than polymer donor/*n*-OS acceptor system (Yang et al., 2017; Shi et al., 2018). In order to further improve the performance of SM-OSCs, it is crucial to deeply investigate the relationship between the molecular structures and device performance of the SM-OSCs.

In the donor-acceptor (D-A) structured *p*-type semiconductor donor materials, benzothiadiazole (BT) unit is a widely used acceptor (A) building block, due to its superior advantages of planar structure and low-lying energy level (Lin and Zhan, 2016). Yan et al. adjusted alkyl chain lengths of BT-based polymer donor (PffBT4T-C_9_C_13_) and the PffBT4T-C_9_C_13_-based OSCs fabricated with a hydrocarbon solvents demonstrate a superior performance (Zhao et al., 2016). Then they designed another BT-based polymer donor P3TEA which demonstrated an efficient OSC with a negligible driving force when blending with a non-fullerene acceptor, SF-PDI_2_ (Liu et al., 2016). Beside polymer donors, BT unit has been widely used in constructing small molecule semiconductor *p*-OS donors. Bazan et al. have systematically investigated BT-based donor-acceptor-donor-acceptor-donor (D-A-D-A-D) type small molecule donor materials (Coughlin et al., 2014). In addition, BT-unit also has been used to construct *n*-type organic semiconductor materials and desirable results have been obtained (Holliday et al., 2016; Baran et al., 2017).

**Figure 1:**
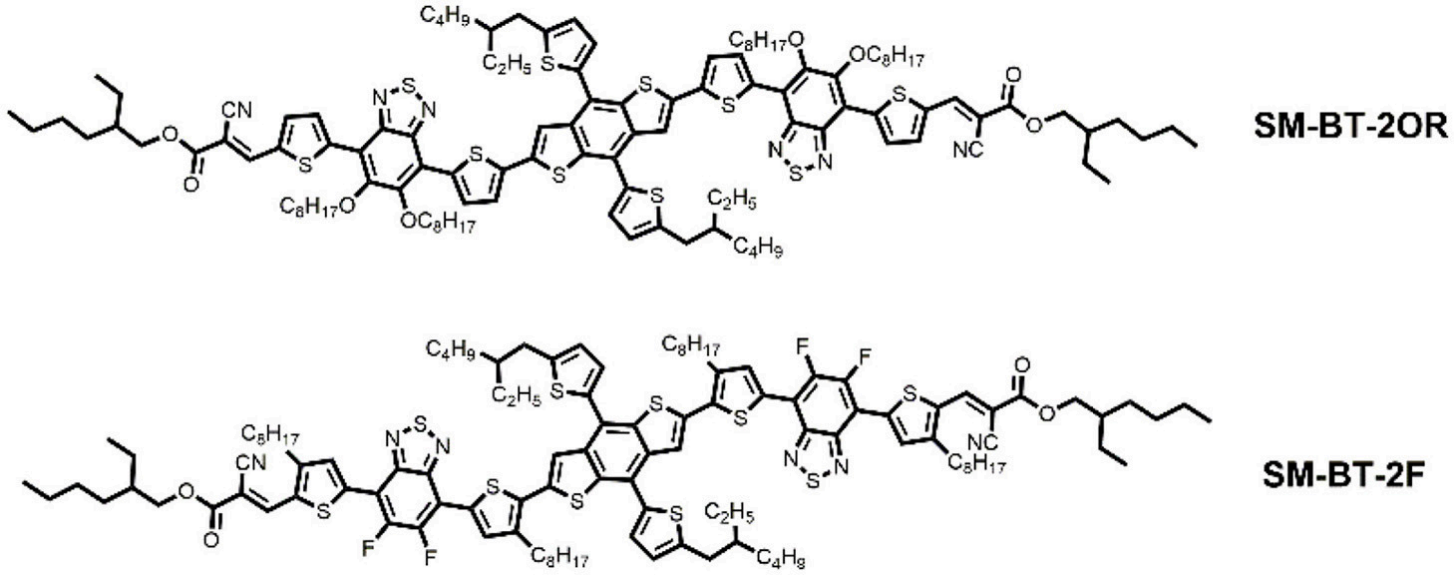
Chemical structures of small molecule donors SM-BT-2OR and SM-BT-2F.

Considering the effective role of side chain engineering in morphology tuning, in order to study the relationship between device performance of the SM-OSCs and molecular structures, herein two BT-based small-molecule donors, SM-BT-2OR and SM-BT-2F (see Figure 1), were designed and synthesized for comparison studies. By rationally introducing alkoxy substituent and fluorine atom (F) on the BT unit, the two small molecules exhibits different absorption characteristics, frontier molecular orbital energy levels, and energy bandgaps. When blending with *n*-type organic semiconductor (*n*-OS) acceptor IDIC, the as cast devices for both small molecules donors showed similar PCE values. The SM-BT-2OR-based devices after thermal annealing (TA) at 120°C for 10 min showed optimized PCE of 7.20% but lower open circuit voltage (*V*_*oc*_), while the SM-BT-2F-based device displayed lower PCE but slightly higher *V*_*oc*_ after the TA treatment. Such opposite trends in the PCE and *V*_oc_ should be ascribed to the different effect of the thermal annealing on the morphology and crystallinity of the two small molecules with the different substituent groups. Our results reveal that for the SM-OSCs the substituent groups have great impact on the film morphology and the photovoltaic performance.

## Results and discussion

### Synthesis and thermal properties

**Scheme 1:**
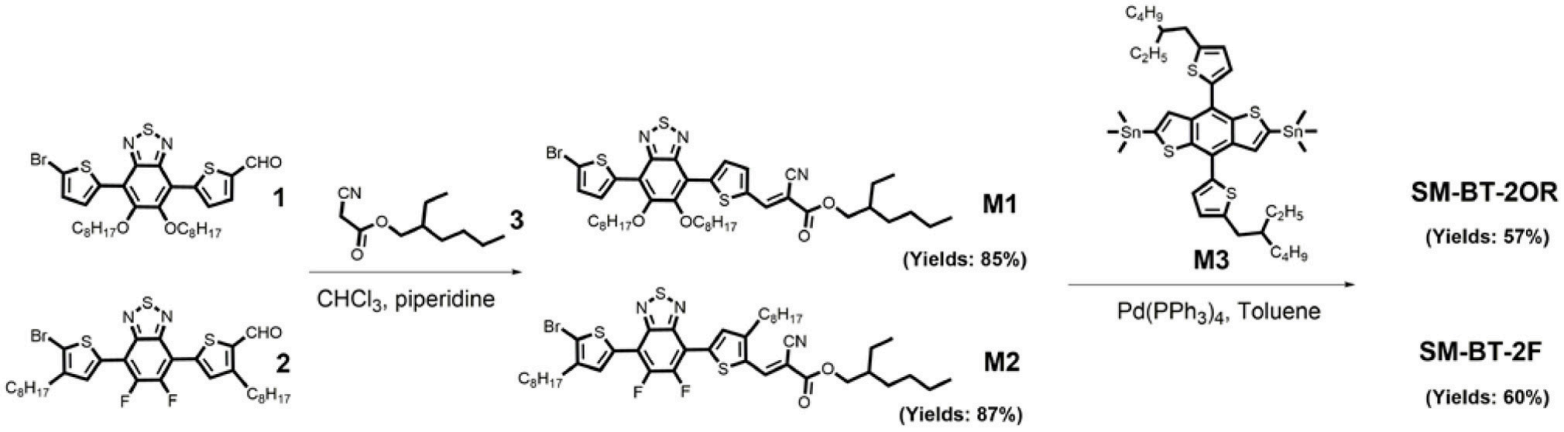
Synthetic routes of small molecule donors SM-BT-2OR and SM-BT-2F.

Scheme 1 shows the synthetic routes of SM-BT-2OR and SM-BT-2F. Compounds **M1** and **M2** were synthesized according to the previously reported methods in high yields (Gu et al., 2012; Feng et al., 2013). Then, the two small molecules SM-BT-2OR and SM-BT-2F were synthesized through similar procedures by a Stille-coupling reaction between compound **M3** and compound **M1** or **M2** with toluene as the solvent and Pd(PPh_3_)_4_ as catalyst. Then, the two molecules were obtained through column chromatography. The two molecules show onset temperatures with 5% weight-loss of 331 and 377°C for SM-BT-2OR and SM-BT-2F, respectively, in the thermogravimetric analysis as shown in Figure S1, indicating that both molecules possess good thermal stability for application in OSCs.

### Photophysical properties and electronic energy levels

**Figure 2:**
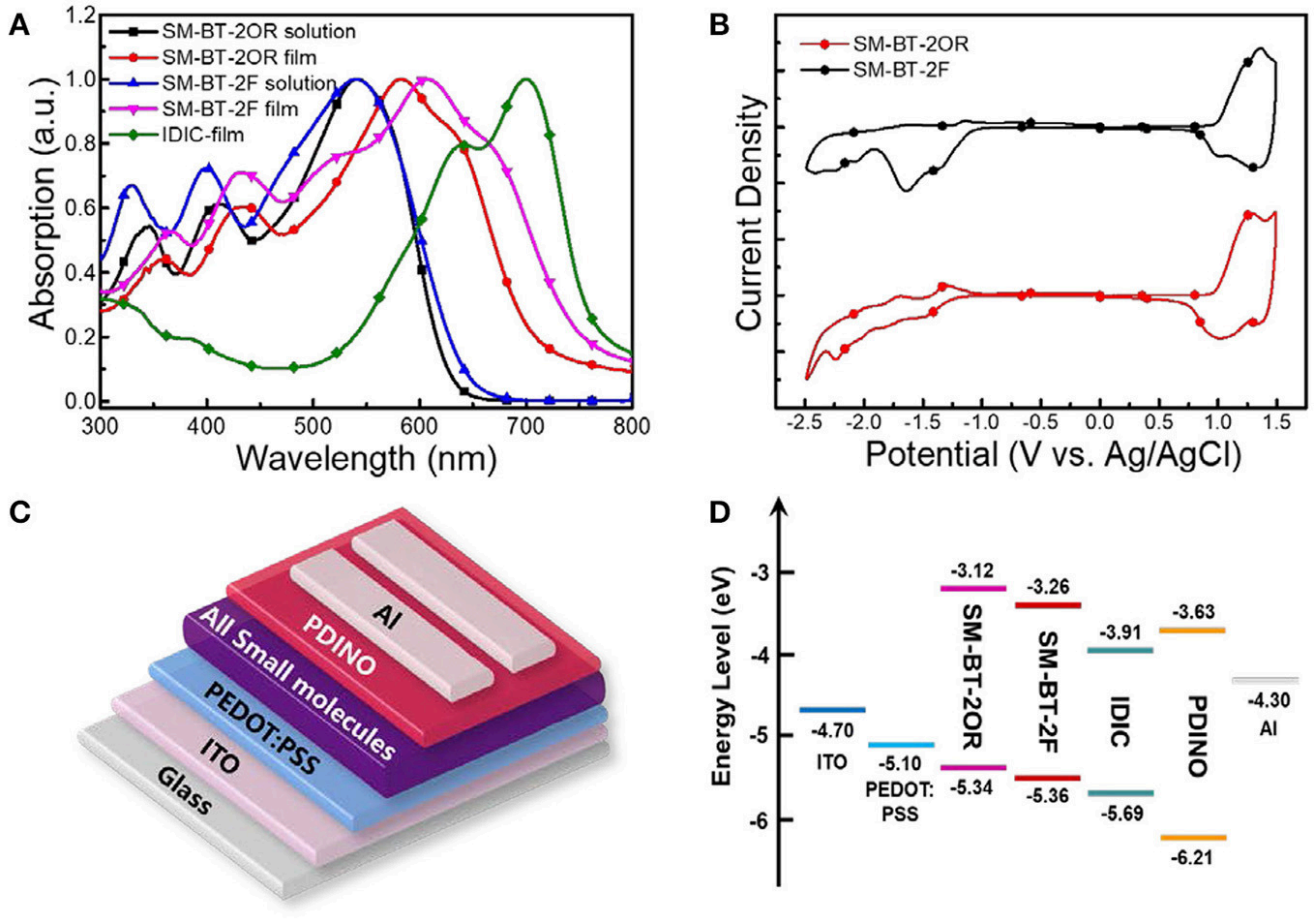
**(A)** Absorption spectra of SM-BT-2OR, SM-BT-2F and IDIC. **(B)** Cyclic voltammograms of SM-BT-2OR and SM-BT-2F. **(C)** Device structure of the SM-OSCs. **(D)** Schematic energy diagram of the materials involved in the SM-OSCs.

Figure 2A shows the UV-vis absorption spectra of the two molecules in dilute CHCl_3_ solutions and as thin films, and IDIC film. The specific absorption characteristics of the two molecules are summarized in Table 1. The two molecules in solution exhibit similar absorption profiles with the same maximum absorption wavelength at 542 nm besides the slightly higher absorption at short wavelength for SM-BT-2F solution. Whereas, in solid films, the maximum absorption wavelength of SM-BT-2F (606 nm) is red-shifted by ~22 nm compared to that of SM-BT-2OR (584 nm), indicating that the introduction of fluorine (F) substituents could influence the stacking behavior of the molecules (Umeyama et al., 2013; Liu et al., 2014; Do et al., 2016). Because of the stronger intermolecular interactions in film state, the maximum absorption peaks of the two molecules show obvious bathochromic shifts of 42 and 64 nm for SM-BT-2OR and SM-BT-2F, respectively. The optical bandgaps ($E_{g}^{opt}$) estimated from the UV-vis absorption onsets (701 nm for SM-BT-2OR and 745 nm for SM-BT-2F) in the film state are determined to be 1.77 and 1.66 eV for SM-BT-2OR and SM-BT-2F, respectively.

**Table 1:** Absorption properties and electronic energy levels of SM-BT-2OR and SM-BT-2F.

**Small molecule**	**λ_max_ solution**	**λ_max_ film**	**λ_onset_ film**	**$E_{\text{g}}^{\text{opt}}$**	***E*_ox_**	***E*_re_**	***E*_HOMO_**	***E*_LUMO_**
	**(nm)**	**(nm)**	**(nm)**	**(eV)**	**(V)**	**(V)**	**(eV)**	**(eV)**
SM-BT-2OR	542	584	701	1.77	0.98	−1.24	−5.34	−3.12
SM-BT-2F	542	606	745	1.66	1.00	−1.10	−5.36	−3.26

The highest occupied molecular orbital (HOMO) and the lowest unoccupied molecular orbital (LUMO) levels of the two molecules were measured by electrochemical cyclic voltammetry with Ag/AgCl as reference electrode, as shown in Figure 2B. The HOMO/LUMO energy levels (*E*_HOMO_/*E*_LUMO_) were calculated from the onset oxidation / reduction potentials (ϕ_ox_/ϕ_red_) according to the equations of *E*_HOMO_/*E*_LUMO_ = –e(ϕ_ox_/ϕ_red_ + 4.8 – ϕ_Fc/Fc+_) (eV) (Bin et al., 2016a). ϕ_Fc/Fc+_ was measured to be 0.44 V vs. Ag/AgCl in this measurement system, and then the calculation equations are *E*_HOMO_/*E*_LUMO_ = –e(ϕ_ox_/ϕ_red_ + 4.36) (eV). As shown in Figure 2B, the onset oxidation potentials (ϕ_ox_) / onset reduction potentials (ϕ_red_) for SM-BT-2OR and SM-BT-2F are 0.98/−1.24 V and 1.00/−1.10 V vs. Ag/AgCl, respectively. The *E*_LUMO_/*E*_HOMO_ of SM-BT-2OR and SM-BT-2F were calculated to be −3.12/−5.34 and −3.26/−5.36 eV, respectively. In comparison with SM-BT-2OR, the *E*_HOMO_ of SM-BT-2F with F atoms substitution is slightly down-shifted by 0.02 eV, which is beneficial for higher *V*_oc_ of the SM-OSCs. Compared with the energy level of IDIC, the LUMO energy level offsets (Δ*E*_LUMO_) and HOMO energy level offsets (Δ*E*_HOMO_) of SM-BT-2OR/IDIC and SM-BT-2F/IDIC are 0.71/0.35 and 0.57/0.33 eV, respectively, which is sufficient for charge separation (Hendriks et al., 2016).

### Photovoltaic performance

**Figure 3:**
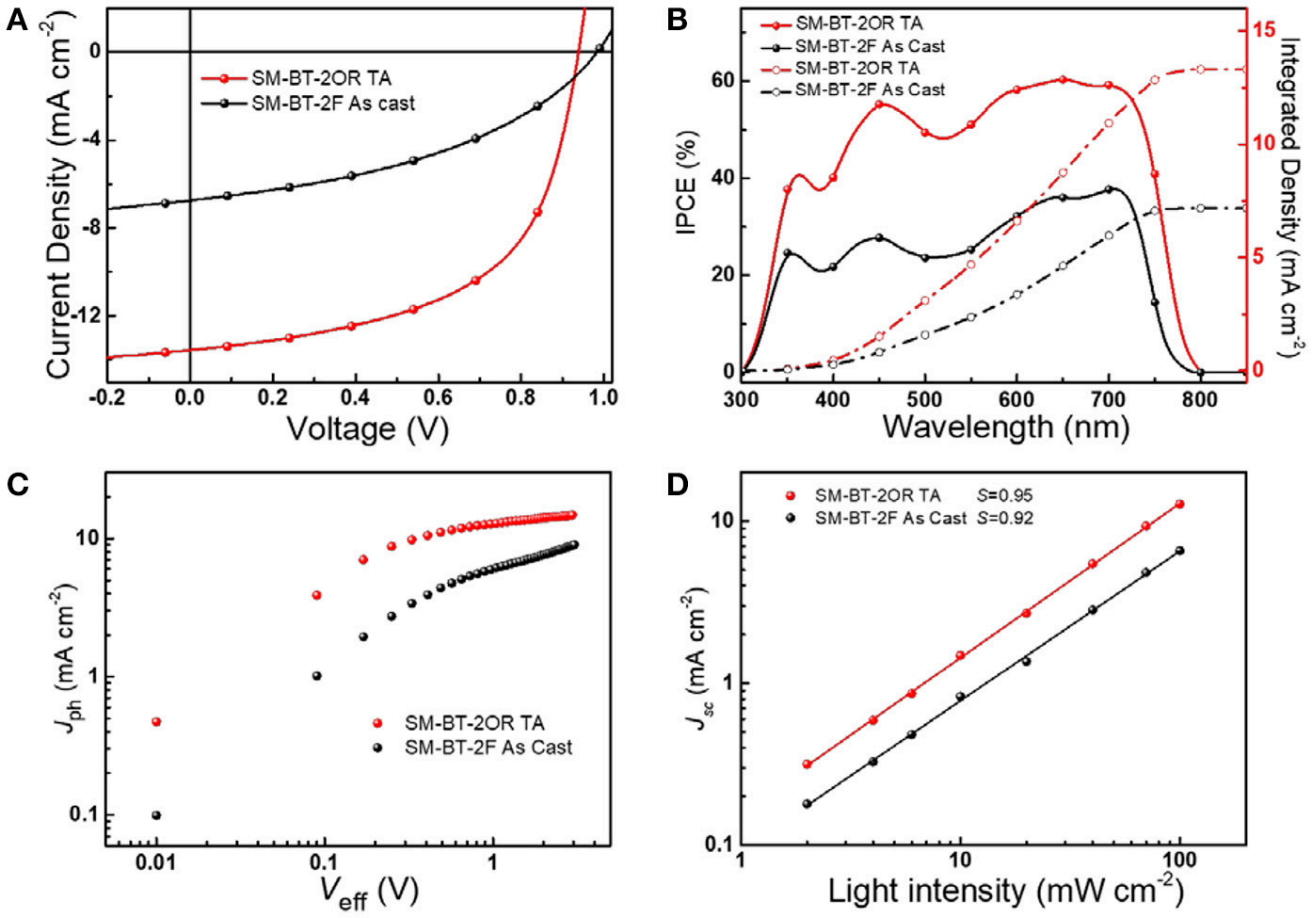
**(A)** *J–V* characteristics of the best SM-OSCs with D/A weight ratio of 2:1 under the illumination of AM 1.5G, 100 mW cm^−2^. **(B)** IPCE spectra of the corresponding devices. **(C)** *J*_ph_ vs. *V*_*eff*_ of the devices. **(D)** Light intensity dependence of *J*_*sc*_ of the devices.

SM-OSCs were fabricated with a conventional device structure of ITO/PEDOT:PSS/*p*-OS:IDIC/PDINO/Al, and characterized to investigate the photovoltaic properties of the *p*-OS SM-BT-2OR and SM-BT-2F (Zhang et al., 2014). Figure 3A shows the typical current density-voltage (*J-V*) curves of the best devices based on SM-BT-2OR and SM-BT-2F under the illumination of AM 1.5G, 100 mW cm^−2^, and the corresponding device performances, including *V*_*oc*_, *J*_*sc*_, FF and PCE, are summarized in Table 2. As shown in Figure 3A, for the as-cast devices, the device based on SM-BT-2OR: IDIC displayed an inferior PCE of 2.33%, with a high *V*_*oc*_ of 0.962 V, but a low *J*_*sc*_ and low FF. The SM-BT-2F-based device showed slightly higher PCE of 2.76%, with a higher *V*_*oc*_ of 0.983 V, which should be ascribed to the deeper HOMO level of SM-BT-2F. Thermal annealing (TA) treatment has been proved to be an effective method to tune the morphology and phase separation of non-fullerene OSCs (Bin et al., 2016b; Chen et al., 2017). Table S1 displays the photovoltaic parameters of the OSCs based on SM-BT-2OR: IDIC (w/w, 2:1) and SM-BT-2F: IDIC (w/w, 2:1) as cast or with TA treatment at different temperatures. Interestingly, for the devices based on SM-BT-2OR: IDIC, TA treatment leads to higher photovoltaic performance, however, for SM-BT-2F: IDIC, TA treatment leads to poorer efficiency. As shown in Table 2, when treated at 120°C for 10 min, the device based on SM-BT-2OR: IDIC demonstrated preferable PCE of 7.20%, with a slightly lower *V*_*oc*_ of 0.939 V, an enhanced *J*_*sc*_ of 13.57 mA cm^−2^ and a higher FF of 56.5%. However, the device based on SM-BT-2F: IDIC showed worse PCE of 1.82%, with a slightly higher *V*_*oc*_ of 0.987 V, but a lower *J*_*sc*_ of 5.36 mA cm^−2^ and a poorer FF of 30.2%. The reverse trends of the TA treatment on the PCE and *V*_oc_ of the devices could be related to the different substituents of the donor molecules.

**Table 2:** Photovoltaic performance parameters of the optimized SM-OSCs based on SM-BT-2OR: IDIC and SM-BT-2F:IDIC.

**Active layer**	**Condition**	***V_*oc*_* (V)**	***J_*sc*_* (mA cm^−2^)**	**FF (%)**	**PCE (%)^a^**	**Slope (*J_*sc*_*)^b^**	**Slope (*V_*oc*_)*^c^**
SM-BT-2OR:IDIC	As cast	0.962	7.10	34.1	2.33 (2.20 ± 0.15)	0.926	1.20 kT/q
	120°C 10 min	0.939	13.57	56.5	7.20 (6.86 ± 0.18)	0.953	1.11 kT/q
SM-BT-2F:IDIC	As cast	0.983	6.74	41.7	2.76 (2.62 ± 0.15)	0.924	1.41 kT/q
	120°C 10 min	0.987	5.36	30.2	1.60 (1.54 ± 0.08)	0.914	1.78 kT/q

a *Average values with standard deviations were obtained from 10 devices*.

b *The slope of the dependence of J_sc_ on light intensity (P) on logarithmic coordinates*.

c *The dependence of V_oc_ on light intensity on semilogarithmic coordinate*.

The input photon to converted current efficiency (IPCE) spectra of the best devices are shown in Figure 3B. Both of the two small molecule-based devices demonstrate broad photo-response from 300 to 800 nm, which indicates that both the small molecule donors and the IDIC acceptor make contributions to the photo current. Compared to the device based on SM-BT-2F, the SM-BT-2OR-based devices present higher photo-response. Especially, the SM-BT-2OR-based device with the TA treatment present a broad plateau with IPCE values of around 50–60% in the wavelength range of *ca*. 420–740 nm, resulting in the relatively higher *J*_*sc*_ of 13.57 mA cm^−2^. The *J*_*sc*_ values of the OSCs based on SM-BT-2OR and SM-BT-2F calculated from integration of the EQE spectra with the AM 1.5G reference spectrum are 13.31 and 7.17 mA cm^−2^, respectively, which are in good agreement with *J*_*sc*_ values measured from *J-V* curves.

In order to investigate the charge dissociation of the SM-OSCs, the relationship between photocurrent density (*J*_*ph*_, *J*_*ph*_ = *J*_*L*_ – *J*_*D*_, where *J*_*L*_ and *J*_*D*_ represent the current densities under the illumination and in the dark, respectively) and effective applied voltage (*V*_*eff*_) was analyzed (Mihailetchi et al., 2004; Wu et al., 2011). As can be seen from Figure 3C, when *V*_*eff*_ arrives at ~3 V, *J*_ph_ value for the SM-BT-2OR-based device with the TA treatment reached saturation (*J*_*sat*_). While for the SM-BT-2F-based device (with and without the TA treatment) and the SM-BT-2OR-based device without the TA treatment (Figure S2), the Jph values do not show a saturation trend within the whole measurement region, suggesting serious recombination process. The charge dissociation and charge collection probability [P(E, T)] in the devices could be estimated by calculating the value of *J*_*ph*_/*J*_*sat*_. Under the short circuit and maximal power output conditions, for the SM-BT-2OR-based devices, the P(E, T) values are 63% and 36% for the as-cast device, and 86 and 63% for the TA treated devices, suggesting a higher charge collection efficiency after TA treatment. However, the corresponding P(E, T) values of the SM-BT-2F-based devices are 68 and 44% for the as-cast device, and 53 and 30% for the TA treated devices, respectively, which means that the SM-BT-2F-based devices possess serious recombination whether with or without TA treatment, which could be ascribed to poorer morphology of the blend films of SM-BT-2F:IDIC.

To further study the charge recombination behavior in the SM-OSCs system, the dependence of *J*_*sc*_ on light intensity (*P*) was evaluated (Figure 3D; Figure S3). Generally, *J*_*sc*_ is known to follow a power-law dependence with respect to *P*_light_, which can be described as *J*_*ph*_ ∞ *P*^α^ (Schilinsky et al., 2002; Koster et al., 2005). For the SM-BT-2OR-based device, the exponential factor (α) is 0.926 for the as-cast device (Figure S3A) and 0.953 for the device with the TA treatment (Figure 3D), indicating that bimolecular recombination could be effectively suppressed by the TA treatment. However, for the SM-BT-2F-based device with the TA treatment (Figure S3B), the exponential factor (α) is only 0.914, which is slightly lower than that without the TA treatment (α = 0.924, Figure 3D), suggesting the existence of a certain amount of bimolecular recombination whether with or without the TA treatment. Compared to the SM-BT-2F-based device, the SM-BT-2OR-based device with the TA treatment shows higher α value, indicating better charge transport capacity, which agrees well with its better device performance. We also measured the dependence of *V*_*oc*_ on the light intensity to investigate the recombination mechanisms. As shown in Figure S4, for the SM-BT-2OR-based devices, the slopes are 1.20 and 1.11 kT/q for the devices as cast and with the TA treatment respectively, which means the recombination at open circuit is a bimolecular dominated process. While for the SM-BT-2F-based devices with or without the TA treatment, the slopes could be separated into two regions, as shown in Figure S5. Even under higher light intensity, the slopes are 1.41 kT/q and 1.78 kT/q for the devices as cast and with TA treatment, respectively, indicating a combination of monomolecular and bimolecular recombination processes. Besides, the larger value of 1.78 kT/q suggests a more undesirable morphology could be formed when the TA treatment was applied for the SM-BT-2F-based device.

The space-charge limited current models, using hole-only and electron-only devices with architectures of ITO/PEDOT:PSS/active layer/Au and ITO/ZnO/active layer/PDINO/Al, respectively, were applied to measure the hole and electron mobilities of the blends before or after the TA treatment (Figures S6, S7). As shown in Table 3, for both blend films, after the thermal annealing treatment, the charge mobilities (electron and hole mobility) were higher than those of the as cast devices. The hole mobilities of the SM-BT-2OR-based devices were measured to be 2.79 × 10^−5^ and 7.37 × 10^−5^ cm^2^ V^−1^ s^−1^ for the devices as cast and with TA treatment, respectively. Compared with the SM-BT-2OR-based devices, the hole mobility of the SM-BT-2F-based blends were rather low whether before (0.37 × 10^−5^ cm^2^ V^−1^ s^−1^) or after the TA treatment (1.77 × 10^−5^ cm^2^ V^−1^ s^−1^), leading to inferior FF and photovoltaic performance. For the SM-BT-2OR-based devices, after the TA treatment, the ratio of hole mobility/electron mobility was slightly balanced, which might benefit for obtaining higher FF of the SM-BT-2OR-based devices. Though the device performance of the SM-BT-2OR-based device with the TA treatment is fairly good, compared to previous results of the SM-OSCs (Qiu et al., 2017; Yang et al., 2018), the hole mobilites of the SM-BT-2OR-based blend is slightly lower, which limits its further improvement of photovoltaic performance.

**Table 3:** Hole and electron mobilities of the SM-BT-2OR: IDIC and SM-BT-2F: IDIC blend films with or without thermal annealing treatment at 120°C for 10 min.

**Active layer**	**Treatment**	**Hole mobility (μ_h_) (cm^2^ V^−1^ s^−1^)**	**Electron mobility (μ_e_) (cm^2^ V^−1^ s^−1^)**	**μ_h_/μ_e_**
SM-BT-2OR:IDIC	As cast	2.79 × 10^−5^ cm^2^ V^−1^ s^−1^	1.26 × 10^−4^ cm^2^ V^−1^ s^−1^	0.22
	120°C 10 min	7.37 × 10^−5^ cm^2^ V^−1^ s^−1^	2.79 × 10^−4^ cm^2^ V^−1^ s^−1^	0.26
SM-BT-2F:IDIC	As cast	0.37 × 10^−5^ cm^2^ V^−1^ s^−1^	0.51 × 10^−4^ cm^2^ V^−1^ s^−1^	0.07
	120°C 10 min	1.77 × 10^−5^ cm^2^ V^−1^ s^−1^	2.19 × 10^−4^ cm^2^ V^−1^ s^−1^	0.08

**Figure 4:**
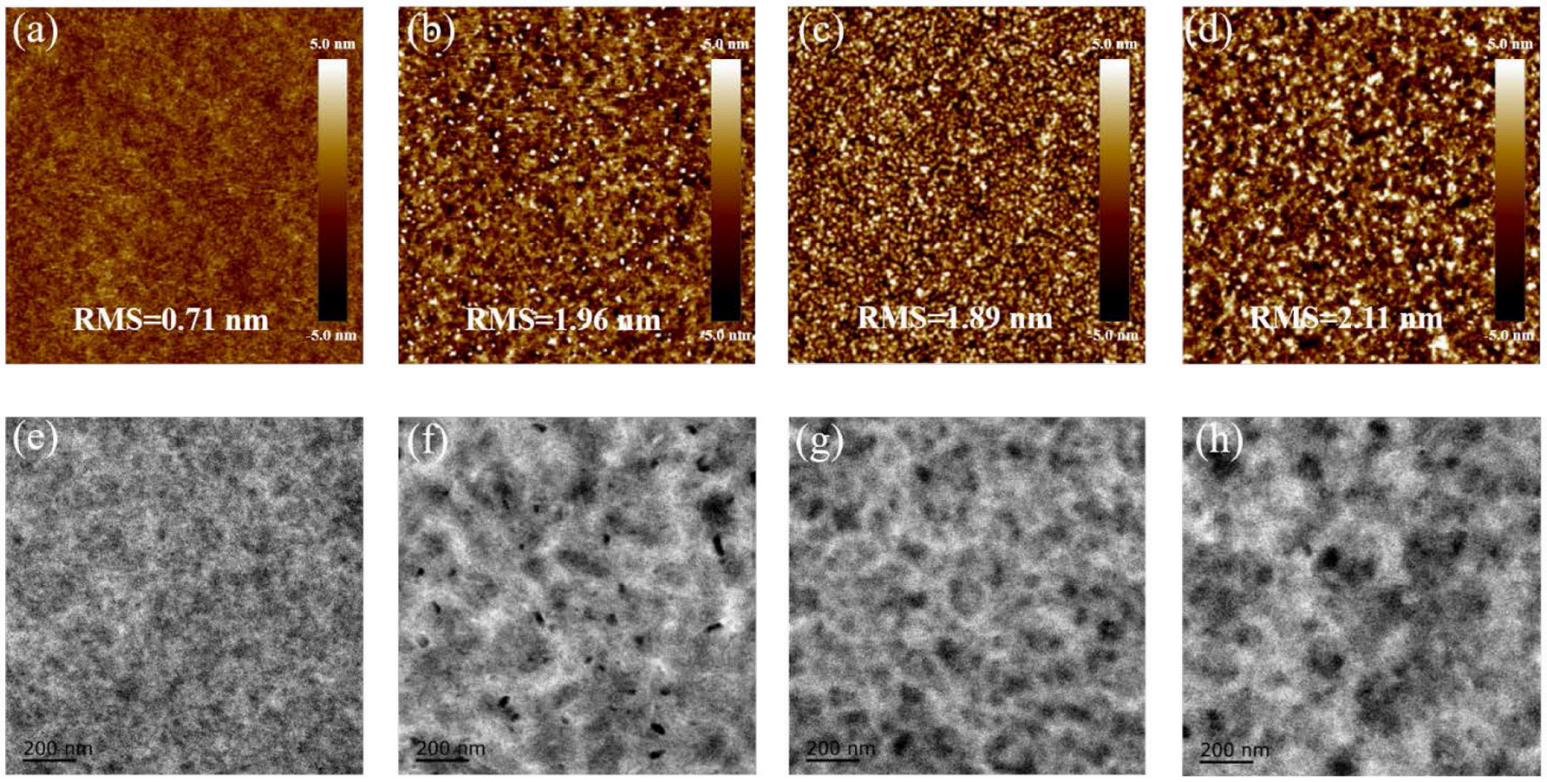
AFM height images (size: 5 × 5 μm^2^) of SM-BT-2OR: IDIC blends as cast **(a)** and TA treated **(b)**, SM-BT-2F: IDIC blends as cast **(c)** and TA treated **(d)**. Root-mean-square (RMS) roughness values are given to describe the smoothness of the morphology. TEM images of SM-BT-2OR: IDIC blends as cast **(e)** and TA treated **(f)**, SM-BT-2F: IDIC blends as cast **(g)** and TA treated **(h)**.

Considering that the device performance is closely related to the blend morphology, detailed investigations on the morphological characteristics of the blend films were carried out (Li et al., 2005; Rivnay et al., 2012). Atomic force microscopy (AFM) was utilized to probe the surface morphologies of the blend films with or without the TA treatment. As shown in Figure 4, all the blends display homogeneous surfaces with a moderate root-mean-square (RMS) roughness. The SM-BT-2F: IDIC blend without the TA treatment presents higher RMS value than that of the SM-BT-2OR: IDIC blend, which should be ascribed to the better crystallinity of SM-BT-2F. Compared to the blend without the TA treatment, the blends with the TA treatment show larger RMS, indicating that the TA treatment could effectively tune the morphology of the active layers of the SM-OSCs. In order to clearly understand aggregation situation of both small molecules in the blend films, transmission electron microscope (TEM) was applied to investigate the phase separation. As shown in Figure 4, the black and white regions in the TEM images of the as-cast blend of SM-BT-2OR: IDIC is uniformly distributed, indicating that SM-BT-2OR and IDIC can be well-mixed. For the as cast blend of SM-BT-2F: IDIC, the TEM image shows relatively large aggregation, which explains the rather low short circuit current density and photovoltaic performance. After thermal annealing, the phase separation of the SM-BT-2F: IDIC blend become even larger, resulting to the more severe recombination, thus leading to the poorer *J*_*sc*_ and device performance. While for the SM-BT-2OR: IDIC blend with the TA treatment, the TEM image shows enhanced phase separation with small fiber like aggregation, which is beneficial for effective exciton dissociation and charge transport, therefore, better device performance could be achieved. In addition, it should be noticed that, although the AFM and TEM images of the SM-BT-2F-based films seem rather uniform, the image of optical microscope presented obvious striped aggregation, as shown in Figure S8, indicating the inferior blend morphology on micrometer scale.

**Figure 5:**
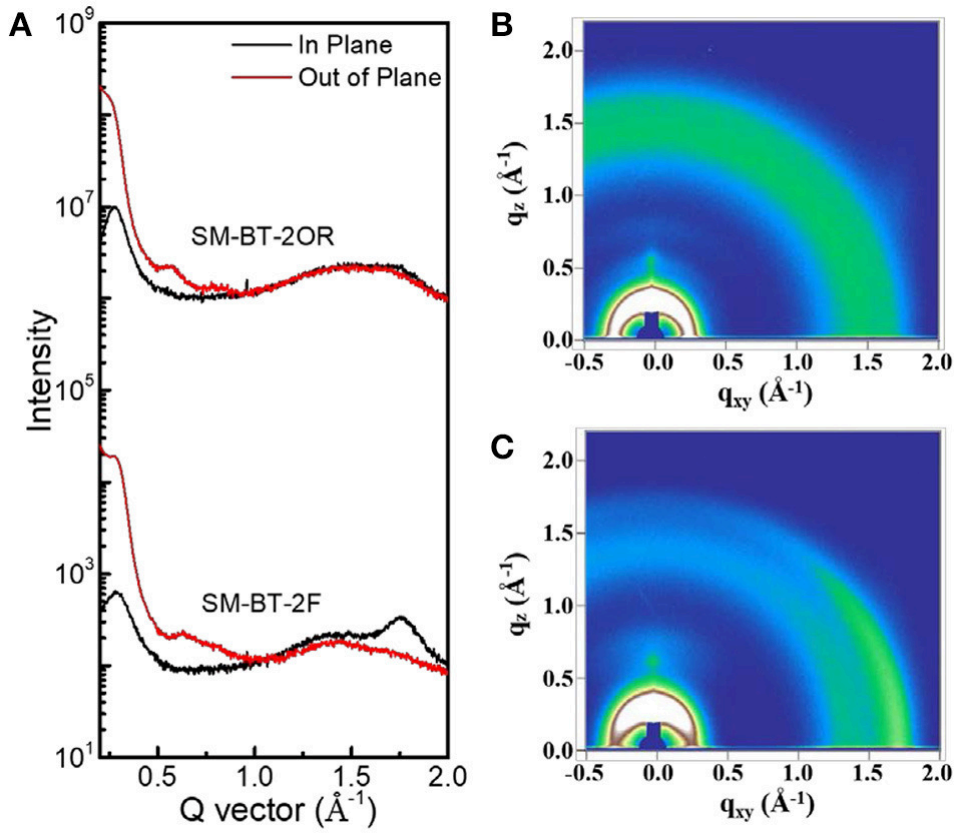
**(A)** Line cuts of the GIWAXS images of neat SM-BT-2OR and SM-BT-2F film; The GIWAXS images of **(B)** neat SM-BT-2OR film and **(C)** neat SM-BT-2F film.

**Figure 6:**
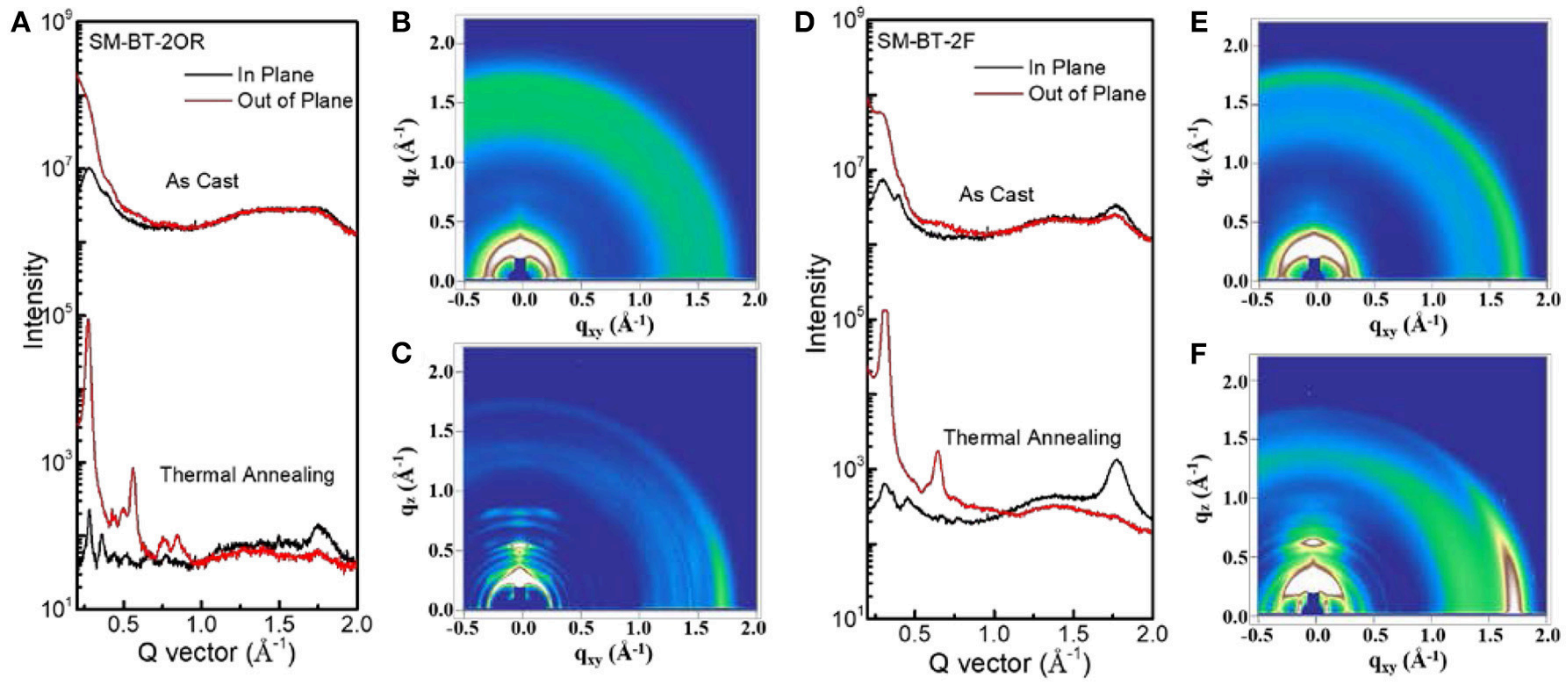
**(A)** Line cuts of the GIWAXS images of SM-BT-2OR: IDIC blend. The GIWAXS images of SM-BT-2OR: IDIC blend **(B)** as cast, **(C)** TA treated. **(D)** Line cuts of the GIWAXS images of SM-BT-2F: IDIC blend. The GIWAXS images of SM-BT-2F: IDIC blend **(E)** as cast and **(F)** TA treated.

To gain deep insight into the molecular stacking differences between SM-BT-2OR and SM-BT-2F, grazing incidence wide-angle X-ray scattering (GIWAXS) measurements were employed (Lilliu et al., 2011; Rivnay et al., 2012; Huang et al., 2014). The 2D GIWAXS patterns and corresponding cut-line profiles in the in-plane and out-of-plane directions are shown in Figure 5 (neat films) and Figure 6 (blend films). The neat SM-BT-2OR films showed amorphous feature with π-π stacking peak at 1.74 Å^−1^ (d-spacing: 3.61 Å) and coherence length of 25.4 Å. The neat SM-BT-2F film displayed edge-on orientations with π-π stacking peak at 1.76 Å^−1^ (*d*-spacing: 3.56 Å) and coherence length of 36.0 Å. The smaller π-π stacking peak and longer coherence length of neat SM-BT-2F film should be ascribed to the better molecular planarity and crystallinity caused by the introduction of F atoms on the BT unit, leading to rather large phase separation as discussed in TEM section.

When blending with IDIC, both films exhibits random orientation and disordered microstructure features with relatively weak peak intensities. After the thermal annealing treatment, significantly stronger peaks with more narrow width accompany by the appearance of new peaks. For both blends of SM-BT-2OR: IDIC and SM-BT-2F: IDIC, the 2D GIWAXS patterns exhibit strong lamellar (100) and (200) diffraction peaks in the out-of-plane direction, indicating a high degree of molecular ordering. Besides, the (010) diffraction peaks demonstrated in-plane preferred orientation, with enhanced coherence length of 47.0 and 52.1 Å, for the SM-BT-2OR: IDIC and SM-BT-2F: IDIC blends, respectively, resulting in better charge transport. Compared to the TA treated blend films of SM-BT-2OR, it can be observed that the SM-BT-2F-based blend film are more prone to adopt a predominant edge-on crystalline orientation, which suggests that it possesses less three-dimensional (3-D) charge pathways in the active layer (Bin et al., 2017; Kumari et al., 2017). Thus, although the molecular packing and phase separation was enhanced, carrier collection was hampered, resulting in lower FF, *J*_*sc*_ and photovoltaic performance. These results indicate that for both small molecules, TA treatment could effectively enhance the molecular packing and affect the molecular orientation in the blend film. In addition, we want to mention that the GIWAXS results show weak face-on stacking, which is also observed for other SM-OSCs based on small molecule donor and small molecule acceptor (Bin et al., 2017; Yang et al., 2017). In order to further improve photovoltaic performance of the SM-OSCs, we should do more work on the morphology optimization of the blend active layer of the p-type small molecule donor and n-type small molecule acceptor.

It should also be mentioned that differing from most other fluorine-containing donor materials SM-BT-2F based device displayed inferior FF and photovoltaic performance, which could be ascribed to the too large phase separation and unsatisfactory molecular orientation of SM-BT-2F.

## Conclusions

In summary, two benzothiadiazole based small-molecule donors, SM-BT-2OR and SM-BT-2F, were designed and synthesized for investigating the effect of the substituents on the photovoltaic performance of the molecules. Compared to SM-BT-2OR, because of the substitution of fluorine atom (F), SM-BT-2F presented red-shifted absorption profile in film state and deeper HOMO level of 5.36 eV. When blending with *n*-type organic semiconductor (*n*-OS) acceptor IDIC, the as-cast devices displayed similar PCE values of 2.33 and 2.76% for the SM-BT-2OR and SM-BT-2F-based devices, respectively. When TA treatment at 120°C for 10 min was applied, the SM-BT-2OR-based devices showed better performance of 7.20%, while the SM-BT-2F-based device displayed even lower PCE. The lower PCE of the SM-BT-2F-based device should be ascribed to the rather large phase separation and more in-plane preferred orientation of π-π stacking when the TA treatment was used, which decreased the exciton dissociation and charge transportation. Besides, the reduced *V*_*oc*_ of the SM-BT-2OR-based devices with the TA treatment should be due to the enhanced phase separation. Our results reveal that for the SM-OSCs the substituent groups have great impact on the film morphology, as well as the photovoltaic performance.

## Author contributions

BQ and YL designed the two small molecules. BQ carried out the materials synthesis and device fabrication and photovoltaic performance studies. Z-GZ, CS, and XL participated in the discussion of the material synthesis. LX and Z-GZ provided the cathode buffer layer material. SC and CY measured the GIWAXS diffraction patterns. YL supervised the project. BQ and YL write the manuscript.

### Conflict of interest statement

The authors declare that the research was conducted in the absence of any commercial or financial relationships that could be construed as a potential conflict of interest.
