# Supplementary material for: Effects of Alkoxy and Fluorine Atom Substitution of Donor Molecules on the Morphology and Photovoltaic Performance of All Small Molecule Organic Solar Cells
Source: Front Chem. 2018 Sep 13;6:413. doi: 10.3389/fchem.2018.00413 (PMC6146101; doi:10.3389/fchem.2018.00413)
Supplement: Supplementary file 1 [file Table_1.DOC]

***Supplementary Material***

**Effects of alkoxy and fluorine atom substitution of donor molecules on the morphology and photovoltaic performance of all small molecule organic solar cells**

***Beibei Qiu,^a b^ Shanshan Chen,^c^ Lingwei Xue,^a^ Chenkai Sun,^a b^ Xiaojun Li,^a b^ Zhi-Guo Zhang,^a^ Changduk Yang, ^c^ Yongfang Li ^a b d^ ****

*^a^* CAS Research/Education Center for Excellence in Molecular Sciences, CAS Key Laboratory of Organic Solids, Institute of Chemistry, Chinese Academy of Sciences, Beijing 100190, China

*^b^* School of Chemical Science, University of Chinese Academy of Sciences, Beijing 100049, China

^c^ Department of Energy Engineering, School of Energy and Chemical Engineering, Low Dimensional Carbon Materials Center, Ulsan National Institute of Science and Technology (UNIST), Ulsan 689-798, South Korea

*^d^* Laboratory of Advanced Optoelectronic Materials, College of Chemistry, Chemical Engineering and Materials Science, Soochow University, Suzhou, Jiangsu 215123, China

*** Corresponding authors:** [liyf@iccas.ac.cn](mailto:liyf@iccas.ac.cn) (Y. Li)

**Experimental Section**

*Materials*

Compound IDIC was commercially available from Solarmer Materials Inc. Pd(PPh_3_)_4_ was obtained from J＆K chemical Co. Compounds 1 and 2 (see Scheme 1) were synthesized according to the previous literatures ^[1-2]^. Toluene was dried over Na/benzophenone and freshly distilled prior to use. The other chemicals, solvents and materials used in this work were all commercially available and used without further purification.

Scheme 1 shows the synthetic routes of **SM-BT-2OR** and **SM-BT-2F.** The detailed synthesis processes are described in the following.

*Synthesis of* ***M1***

Compound **1** (1.33 g, 2 mmol), **3** (1.97 g, 10 mmol) and three drops piperidine were dissolved in 30 mL CHCl_3_. The reaction mixture was purged with argon for 15 min and then stirred at room temperature for 24 h. Solvent was removed under reduced pressure and the residue was purified by silica gel column chromatography using a mixture of petroleum and dichloromethane ether (2:1) as eluent to afford compound **M1** as a red solid (1.43 g, 85%).

^1^H NMR (300 MHz, CDCl_3_): δ 8.65 (d, *J*=4.1 Hz, 1H), 8.40 (d, *J*=4.1 Hz, 1H), 8.19 (s, 1H), 7.85 (d, *J*=4.0 Hz, 1H), 7.18 (d, *J*=4.0 Hz, 1H), 4.23-4.04 (m, 6H), 2.07-1.78 (m, 5H), 1.47-1.15 (m, 28H), 0.95-0.74 (m, 12H). MS (MALDI-TOF): *m/z* 841.5 (M^+^).

*Synthesis of* ***M2***

Compound **2** (1.34 g, 2 mmol), **3** (1.97 g, 10 mmol) and three drops piperidine were dissolved in 30 mL CHCl_3_. The reaction mixture was purged with argon for 15 min and then stirred at room temperature for 24 h. Solvent was removed under reduced pressure and the residue was purified by silica gel column chromatography using a mixture of petroleum and dichloromethane ether (2:1) as eluent to afford compound **M2** as red solid (1.47 g, 87%).

^1^H NMR (300 MHz, CDCl_3_): δ 8.44 (s, 1H), 8.13 (s, 1H), 7.99 (s, 1H), 4.22 (d, *J*=5.7 Hz, 2H), 2.85 (t, *J*=7.5 Hz, 2H), 2.63 (t, *J*=7.5 Hz, 2H), 1.66 (dd, *J*=18.3 Hz, 11.4 Hz, 4H), 1.54-1.16 (m, 28H), 0.89 (dt, *J*=11.8, 7.2 Hz, 12H). MS (MALDI-TOF): *m/z* 845.7 (M^+^).

*Synthesis of* ***SM-BT-2OR***

A solution of **M1** (320 mg, 0.38 mmol) and **4** (135 mg, 0.15 mmol) in dry toluene (12 mL) was degassed several times with argon followed by the addition of Pd(PPh_3_)_4_ (30 mg). After stirring at 110 ^o^C for 36 h under argon, the mixture was poured into water and extracted with CHCl_3_. The residue was purified by silica gel chromatography using a mixture of petroleum and dichloromethane ether (1:1) as eluent to afford compound **SM-BT-2OR** as a black solid (180 mg, 57%).

^1^H NMR (300 MHz, CDCl_3_) δ 8.69 (d, *J*=4.4 Hz, 2H), 8.42 (d, *J*=3.9 Hz, 2H), 8.21 (s, 2H), 7.76 (d, *J*=4.4 Hz, 2H), 7.62 (s, 2H), 7.32 (d, *J*=3.3 Hz, 2H), 7.23 (d, *J*=3.9 Hz, 2H), 6.91 (d, *J*=3.3 Hz, 2H), 4.21-4.03 (m, 12H), 2.88 (d, *J*=6.6 Hz, 4H), 2.08-1.82 (m, 10H), 1.72-1.60 (m, 2H), 1.53-1.13 (m, 72H), 0.98-0.77 (m, 36H).

^13^C NMR (75 MHz, CDCl_3_) δ 163.27, 153.50, 151.29, 150.50, 150.34, 146.27, 145.95, 144.30, 139.68, 138.82, 137.71, 137.40, 136.95, 136.33, 134.10, 132.41, 131.58, 127.95, 125.47, 125.08, 123.40, 119.41, 119.17, 116.05, 115.95, 98.24, 77.23, 75.06, 74.66, 68.81, 41.54, 38.81, 34.45, 32.59, 31.88, 30.47, 30.35, 30.18, 29.61, 29.49, 29.39, 29.36, 28.99, 28.94, 26.02, 25.83, 23.79, 23.12, 22.98, 22.71, 14.25, 14.13, 14.08, 11.04.

MS (MALDI-TOF): *m/z* 2101.9 (M^+^).

Elemental analysis: calculated for C_118_H_152_N_6_O_8_S_10_ (%): C, 67.39; H, 7.29; N, 4.00. Found (%): C, 67.09; H, 7.30; N, 4.16.

*Synthesis of* ***SM-BT-2F***

**SM-BT-2F** was synthesized similar to that described above for the synthesis of **SM-BT-2OR**, and a black solid was obtained (190 mg, 60%).

^1^H NMR (300 MHz, CDCl_3_) δ 8.14 (s, 2H), 7.98 (s, 2H), 7.84 (s, 2H), 7.43-7.33 (m, 4H), 7.02 (d, *J*=2.4 Hz, 2H), 4.12 (d, *J*=4.1 Hz, 4H), 3.10-2.92 (m, 4H), 2.80-2.55 (m, 8H), 1.93-1.79 (m, 2H), 1.78-1.22 (m, 82H), 1.17-0.88 (m, 36H).

^13^C NMR (75 MHz, CDCl_3_) δ 163.09, 153.88, 148.10, 147.97, 145.49, 142.98, 140.38, 139.59, 139.40, 138.02, 137.00, 136.38, 136.11, 132.36, 129.56, 128.25, 125.31, 122.70, 120.68, 115.69, 112.60, 108.96, 97.31, 95.52, 89.35, 77.23, 68.77, 41.56, 38.77, 34.49, 32.74, 32.13, 31.90, 31.27, 30.35, 30.09, 29.73, 29.68, 29.61, 29.44, 29.33, 29.06, 28.91, 25.71, 23.78, 23.22, 23.01, 22.87, 22.71, 14.31, 14.22, 14.13, 14.08, 11.03, 10.98.

MS (MALDI-TOF): *m/z* 2109.9 (M^+^).

Elemental analysis: calculated for C_118_H_148_F_4_N_6_O_4_S_10_ (%): C, 67.14; H, 7.07; N, 3.98. Found (%): C, 67.12; H, 7.09; N, 3.97.

**Material characterization**

^1^H NMR and ^13^C NMR spectra were recorded on Bruker AVANCE 300 MHz NMR spectrometer at room temperature. Mass spectra were measured on a Shimadzu spectrometer. The UV-vis absorption spectra were measured on a Hitachi U-3010 UV-vis spectrophotometer. Cyclic voltammogram (CV) measurements were conducted on a Zahner IM6e electrochemical workstation using sample film coated on glassy carbon as the working electrode, Pt wire as the counter electrode, and Ag/AgCl as the reference electrode in a 0.1 M tetrabutylammonium hexafluorophosphate (Bu_4_NPF_6_) acetonitrile solution. The ferrocene/ferrocenium (Fc/Fc^+^) couple was used as an internal reference.

**Device fabrication and characterization**

The SM-OSCs were fabricated with a structure of ITO/PEDOT:PSS/active layer/PDINO/Al. The PEDOT:PSS aqueous solution (Baytron P 4083 from H. C. Starck) was filtered through a 0.45 mm filter and spin-coated on precleaned ITO-coated glass at 4000 rpm for 30 s. Subsequently, the PEDOT:PSS film was baked at 150 ^o^C for 20 min in air to give a thin film with a thickness of ~30 nm. A blend of the small molecule and IDIC was dissolved in chloroform (CHCl_3_) and spin-coated the blend solutions at 2500 rpm for 30 s onto the PEDOT:PSS layer. The thickness of the photovoltaic layer is in the range of 110~120 nm. Then methanol solution of PDINO at a concentration of 1.0 mg mL^-1^ was deposited atop the active layer at 3000 rpm for 30 s to afford a PDINO cathode buffer layer with thickness of about 10 nm. Finally, the metal cathode Al was thermally evaporated under a shadow mask with a base pressure of approximately 10^-5^ Pa. The photovoltaic area of the device is 4.5 mm^2^. Optical microscope (Olympus BX51) was used to define the active area of the devices. The (current density-voltage) *J-V* characteristics of the SM-OSCs were measured in a nitrogen glove box with a Keithley 2450 Source Measure unit. Oriel Sol3A Class AAA Solar Simulator (model, Newport 94023A) with a 450W xenon lamp and an air mass (AM) 1.5 filter was used as the light source. The light intensity was calibrated to 100 mW cm^-2^ by a Newport Oriel 91150V reference cell. The input photon to converted current efficiency (IPCE) was measured by Solar Cell Spectral Response Measurement System QE-R3-011 (Enli Technology Co., Ltd., Taiwan). The light intensity at each wavelength was calibrated with a standard single-crystal Si photovoltaic cell.


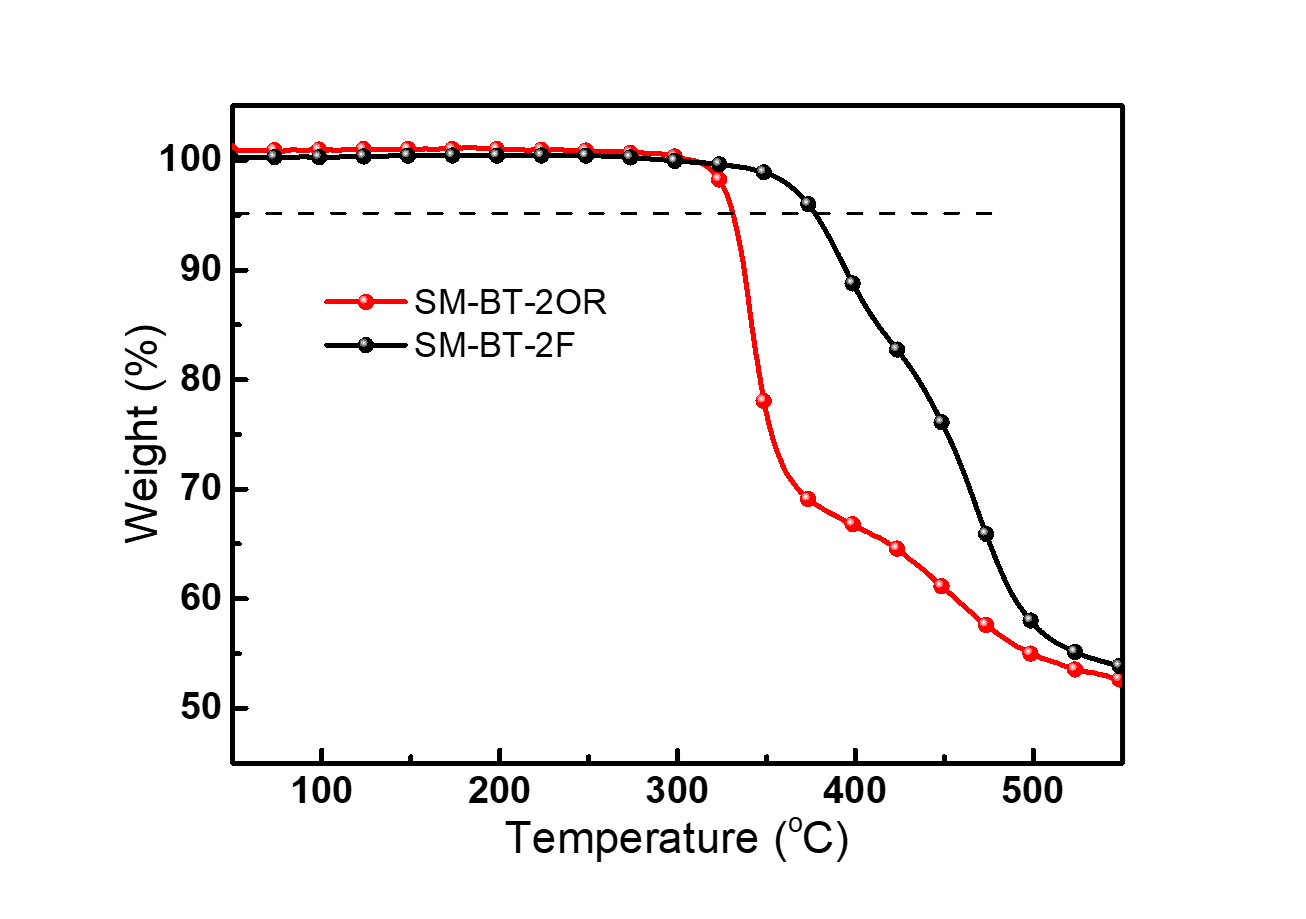


**Figure S1.** TGA plots of SM-BT-2OR and SM-BT-2F with a heating rate of 10 °C min^-1^ under an inert atmosphere.


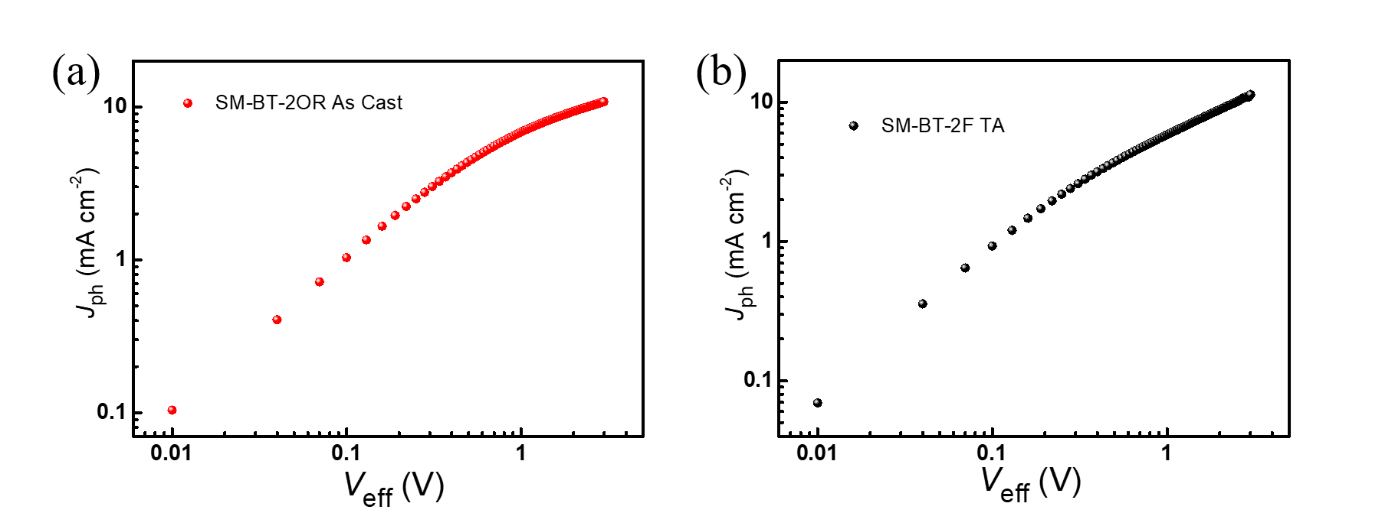


**Figure S2**. *J_ph_* versus *V_eff_* of the devices based on SM-BT-2OR: IDIC (as cast) and SM-BT-2F: IDIC (TA).


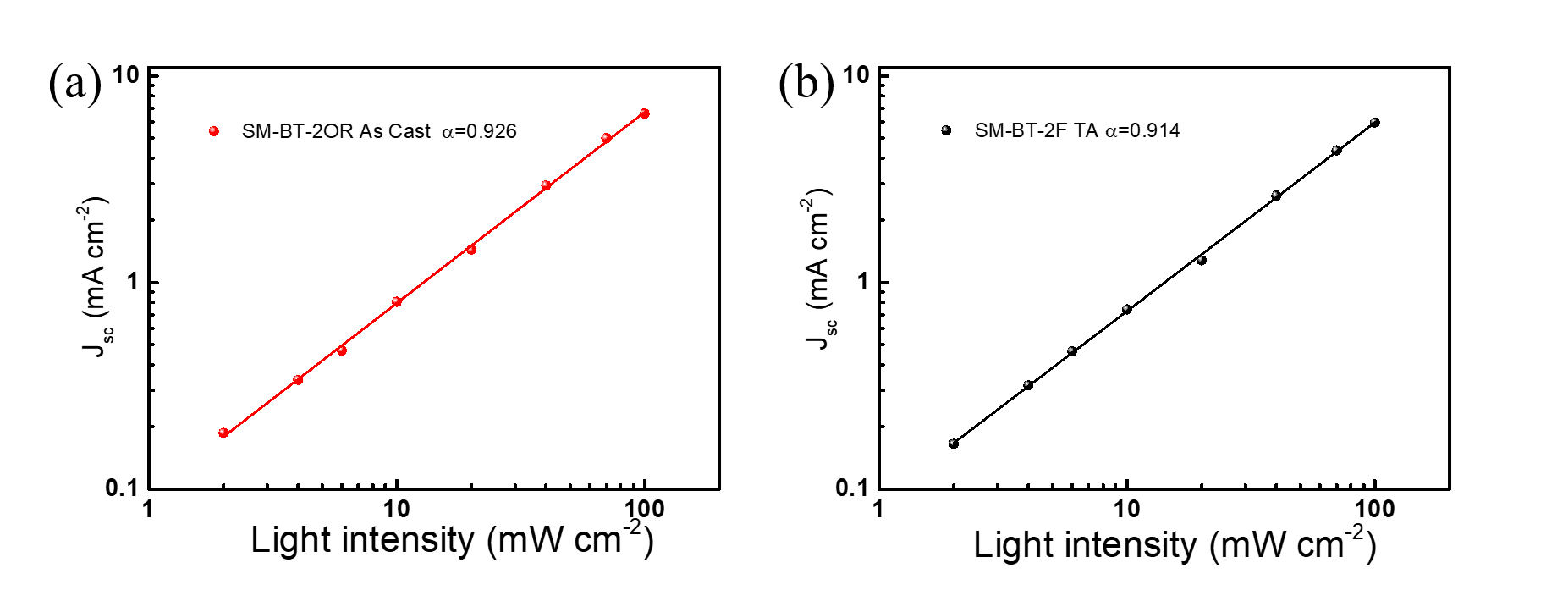


**Figure S3**. Light intensity dependence of *J_sc_* of the devices based on SM-BT-2OR: IDIC (as cast) and SM-BT-2F: IDIC (TA).


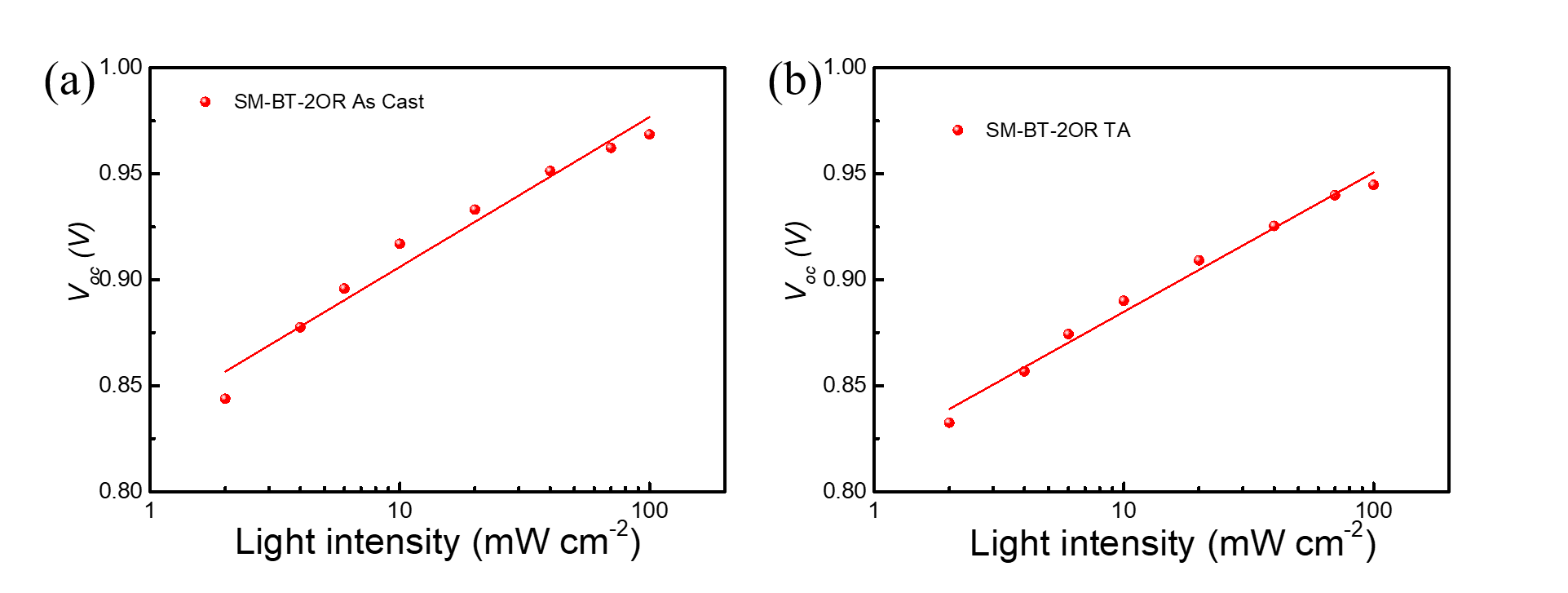


**Figure S4**. Light intensity dependence of *V_oc_* of the devices based on SM-BT-2OR: IDIC as cast or with TA treatment.


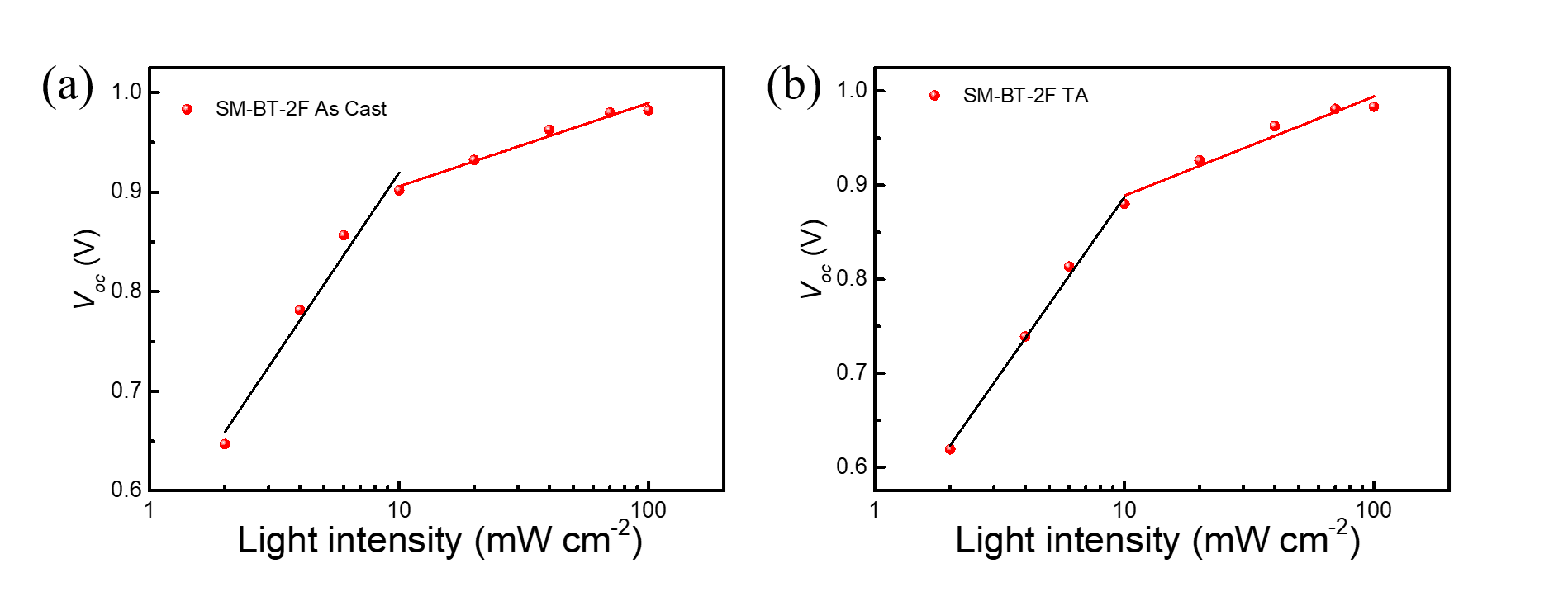


**Figure S5**. Light intensity dependence of *V_oc_* of the devices based on SM-BT-2F: IDIC as cast or with TA treatment.


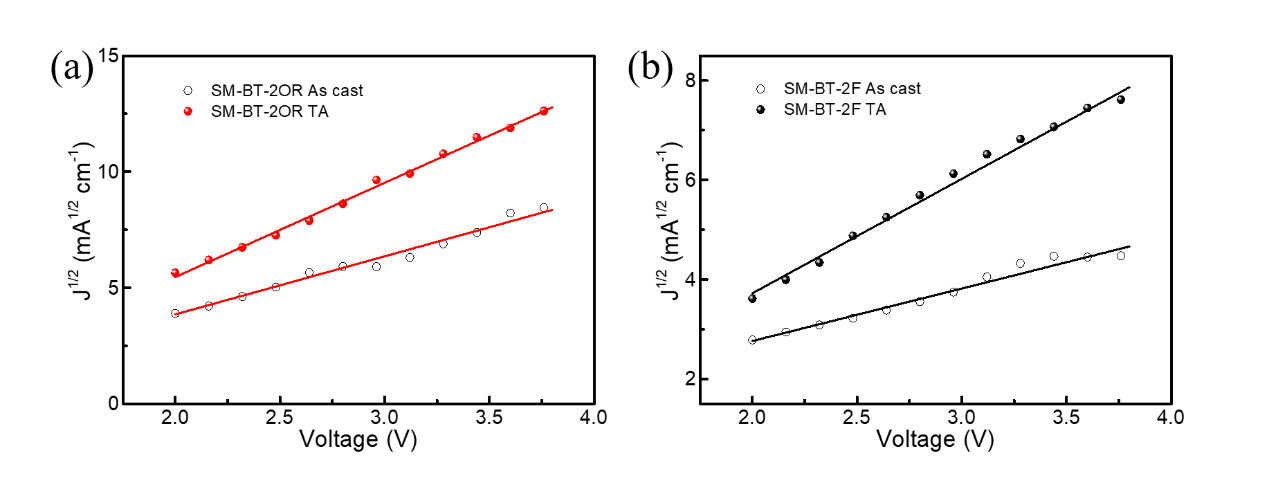


**Figure S6**. *J-V* characteristics in the dark for the hole-only devices based on (a) SM-BT-2OR: IDIC and (b) SM-BT-2F: IDIC with and without thermal annealing treatment at 120 ^o^C for 10.min


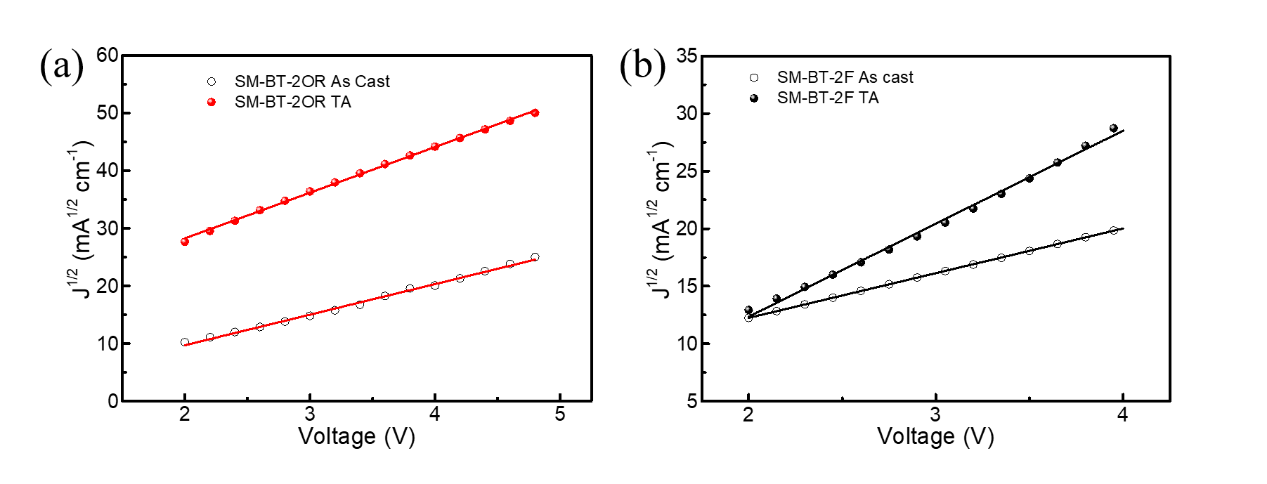


**Figure S7**. *J-V* characteristics in the dark for the electron-only devices based on (a) SM-BT-2OR: IDIC and (b) SM-BT-2F: IDIC with and without thermal annealing at 120 ^o^C for 10.min t.


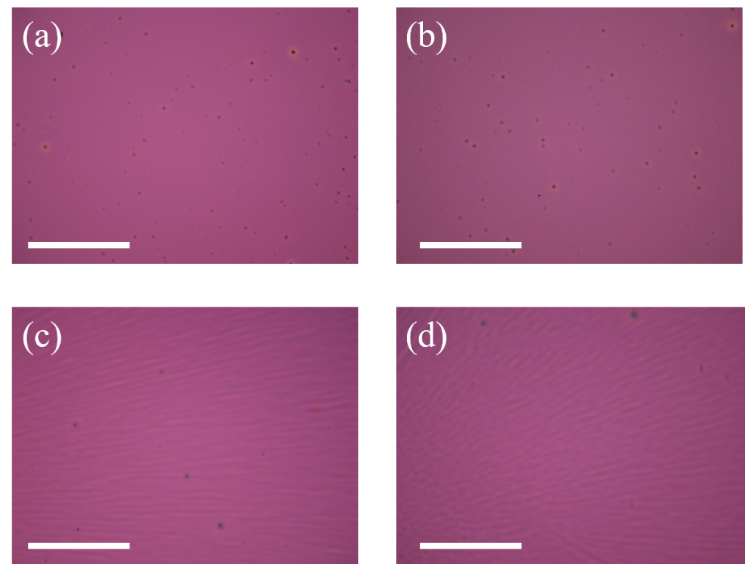


**Figure S8**. Optical microscopy images (scale bar is 500 µm) of the SM-BT-2OR: IDIC and SM-BT-2F: IDIC blends with or without thermal annealing treatment. at 120 ^o^C for 10.min (Bar=500 μm.)

**Table S1.** Photovoltaic properties of the SM-OSCs based on SM-BT-2OR: IDIC (2:1) and SM-BT-2F: IDIC (2:1) with the thermal annealing (TA) treatment at different temperatures

| Active layer | Temperature (^o^C) | *V*_oc_ (V) | *J*_sc_ (mA cm^-2^) | FF (%) | PCE (%) |
| --- | --- | --- | --- | --- | --- |
| SM-BT-2OR:IDIC | Without TA | 0.962 | 7.10 | 34.1 | 2.33 |
|  | 100 | 0.962 | 10.60 | 54.4 | 5.55 |
|  | 110 | 0.957 | 11.92 | 57.5 | 6.57 |
|  | 120 | 0.939 | 13.57 | 56.5 | 7.20 |
|  | 130 | 0.948 | 11.67 | 55.7 | 6.15 |
| SM-BT-2F:IDIC | Without TA | 0.983 | 6.74 | 41.7 | 2.76 |
|  | 100 | 1.002 | 5.92 | 35.5 | 2.10 |
|  | 120 | 0.987 | 5.36 | 30.2 | 1.60 |

**Reference:**

[1] Gu, Z., et al., Synthesis and photovoltaic properties of conjugated side chains polymers with different electron-withdrawing and donating end groups. *J. Polym. Sci., Part A: Polym. Chem.* **2012,** *50* (18), 3848-3858.

[2] Feng, Q., et al., Enhanced performance of quasi-solid-state dye-sensitized solar cells by branching the linear substituent in sensitizers based on thieno[3,4-c]pyrrole-4,6-dione. *Chem. Asian J.* **2013,** *8* (1), 168-177.
